# Weight-dependent and weight-independent effects of dulaglutide on blood pressure in patients with type 2 diabetes

**DOI:** 10.1186/s12933-023-01775-x

**Published:** 2023-03-09

**Authors:** Keith C. Ferdinand, Julia Dunn, Claudia Nicolay, Flora Sam, Emily K. Blue, Hui Wang

**Affiliations:** 1 Tulane University School of Medicine, New Orleans, LA USA; 2 Eli Lilly and Company, Indianapolis, IN USA; 3 TechData Service Company, King of Prussia, PA USA

**Keywords:** Blood pressure, Diabetes, Dulaglutide, Glucagon-like peptide-1, Hypertension, Weight

## Abstract

**Background:**

Patients with type 2 diabetes (T2D) treated with glucagon-like peptide-1 receptor agonists may experience reductions in weight and blood pressure. The primary objective of the current study was to determine the weight-dependent and weight-independent effects of ~ 6 months treatment with dulaglutide 1.5 mg treatment in participants with T2D.

**Methods:**

Mediation analysis was conducted for five randomized, placebo-controlled trials of dulaglutide 1.5 mg to estimate the weight-dependent (i.e., mediated by weight) and weight-independent effects from dulaglutide vs. placebo on change from baseline for systolic blood pressure (SBP), diastolic blood pressure (DBP), and pulse pressure. A random-effects meta-analysis combined these results. To investigate a dose response between dulaglutide 4.5 mg and placebo, mediation analysis was first conducted in AWARD-11 to estimate the weight-dependent and weight-independent effects of dulaglutide 4.5 mg vs. 1.5 mg, followed by an indirect comparison with the mediation result for dulaglutide 1.5 mg vs. placebo.

**Results:**

Baseline characteristics were largely similar across the trials. In the mediation meta-analysis of placebo-controlled trials, the total treatment effect of dulaglutide 1.5 mg after placebo-adjustment on SBP was − 2.6 mmHg (95% CI − 3.8, − 1.5; p < 0.001) and was attributed to both a weight-dependent effect (− 0.9 mmHg; 95% CI: − 1.4, − 0.5; *p* < 0.001) and a weight-independent effect (− 1.5 mmHg; 95% CI: − 2.6, − 0.3; *p* = 0.01), accounting for 36% and 64% of the total effect, respectively. For pulse pressure, the total treatment effect of dulaglutide (− 2.5 mmHg; 95% CI: − 3.5, − 1.5; *p* < 0.001) was 14% weight-dependent and 86% weight-independent. For DBP there was limited impact of dulaglutide treatment, with only a small weight-mediated effect. Dulaglutide 4.5 mg demonstrated an effect on reduction in SBP and pulse pressure beyond that of dulaglutide 1.5 mg which was primarily weight mediated.

**Conclusions:**

Dulaglutide 1.5 mg reduced SBP and pulse pressure in people with T2D across the placebo-controlled trials in the AWARD program. While up to one third of the effect of dulaglutide 1.5 mg on SBP and pulse pressure was due to weight reduction, the majority was independent of weight. A greater understanding of the pleotropic effects of GLP-1 RA that contribute to reduction in blood pressure could support developing future approaches for treating hypertension.

*Trial registrations (clinicaltrials.gov)* NCT01064687, NCT00734474, NCT01769378, NCT02597049, NCT01149421, NCT03495102

**Supplementary Information:**

The online version contains supplementary material available at 10.1186/s12933-023-01775-x.

## Introduction

**Fig. 1:**
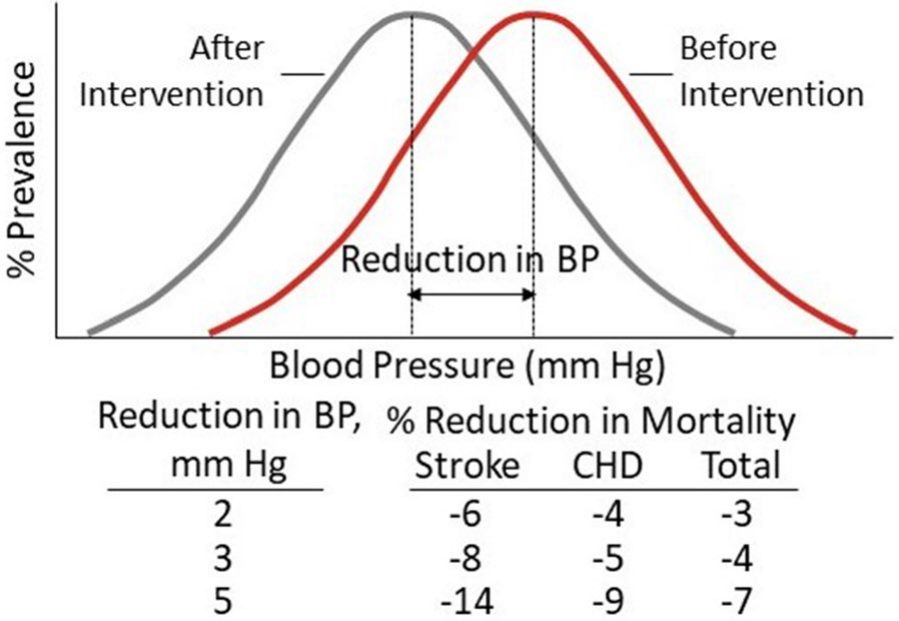
Distribution of systolic blood pressure before and after intervention. *BP* blood pressure, *CHD* coronary heart disease. Adapted from Whelton PK, et al. [6]

Elevated blood pressure (BP) is highly prevalent and is reported in about three-fourths of patients with type 2 diabetes (T2D) [1]. The role of elevated BP in both macrovascular and microvascular complications is well-established in patients with T2D [2, 3] and is recognized as a common and robust predisposing risk factor for cardiovascular disease [4, 5]. Importantly, relatively small reductions in the mean systolic blood pressure (SBP) (2–5 mmHg) are sufficient to reduce cardiovascular events and death in a population (Fig. 1) [6]. In a meta-analysis of recent blood pressure trials, the Blood Pressure Lowering Treatment Trialists’ Collaboration reported that a 5-mmHg reduction in SBP lowers the risk of major cardiovascular events by 10%, and the benefit was independent of the baseline presence of cardiovascular disease [7]. In a large meta-regression analysis of patients with diabetes, the risk of stroke decreased by 13% for each 5-mmHg reduction in SBP and by 11.5% for each 2-mmHg reduction in diastolic blood pressure (DBP) [8]. Similarly, results from the UK Prospective Diabetes Study (UKPDS) showed that among patients with mean SBP ranging from 159–160 mmHg, a 10-mmHg reduction in SBP decreased diabetes-related mortality by 15% and all-cause mortality by 12% [9].

The current American College of Cardiology/American Heart Association multi-society guideline recommends individualized BP treatment targets for patients with T2D based on cardiovascular risk, with a goal of < 130/80 mmHg; individualized targets should account for patient tolerance of the BP level [10]. However, up to half of patients do not achieve these goals [11, 12]. Since small reductions in BP occur with glucagon-like peptide-1 receptor agonist (GLP-1 RA) treatment, it has been proposed that these reductions in BP and the specific mechanisms contributing to BP reduction with GLP-1 RAs may be relevant to decreases in major adverse cardiovascular events (MACE) observed with GLP-1 RA treatment [13, 14]. The specific mechanisms contributing to BP reduction with GLP-1 RAs are unknown, but improvement in arterial stiffness is a probable mechanism [15].

Treatment of patients with T2D with GLP-1 RAs also results in modest weight reduction [16] which may contribute to the long-term reduction in BP, as well as prevent the onset of hypertension [17, 18]. However, previous large metanalyses have reported conflicting results [18, 19]. In a randomized, placebo-controlled clinical trial to characterize the effect of dulaglutide 1.5 mg vs. placebo on BP and heart rate in participants with T2D using 24-h ambulatory BP monitoring, participants who received dulaglutide demonstrated a 2.5 mmHg decrease in 24-h SBP vs. an increase of 0.2 mmHg with placebo at week 26, with limited change in DBP. There was no significant association found between weight reduction and BP reduction occurring with dulaglutide treatment in this single trial [20].

The current study aimed to better characterize the relationship between dulaglutide treatment and changes in BP and pulse pressure in participants with T2D. The primary objective was to determine the weight-dependent and weight-independent effects of treatment through a mediation meta-analysis of the all the dulaglutide placebo-controlled trials. The secondary objective was to investigate the effect of higher dose dulaglutide treatment on the weight-dependent and weight-independent effects of blood pressure change.

## Methods

### Studies

The analyses included six pivotal randomized, double-blind trials of dulaglutide 1.5 mg in participants with T2D that measured sitting SBP and DBP from vital sign data around the timeline of 6 months (week 24 to week 26). Five placebo-controlled studies were used to estimate the effects between dulaglutide 1.5 mg and placebo. AWARD-1 (NCT01064687), AWARD-5 (NCT00734474), AWARD-8 (NCT01769378), and AWARD-10 (NCT02597049) were phase 3, placebo-controlled trials which investigated the safety and glycemic efficacy of dulaglutide with various background glycemic therapies (Table 1). Ferdinand et al. (NCT01149421) was a phase 2, randomized, double-blind, placebo-controlled trial which evaluated BP and heart rate effects of dulaglutide vs. placebo in participants with T2D with and without hypertension and BP < 140/90 mmHg. In addition, AWARD-11 (NCT03495102) was a phase 3, non-placebo-controlled trial to evaluate safety and glycemic efficacy of dulaglutide 3.0 mg and 4.5 mg to dulaglutide 1.5 mg.

**Table 1:** Study design for placebo-controlled trials included in the meta-analysis

Parameters	AWARD-1	AWARD-5	AWARD-8	AWARD-10	AWARD-11	Ferdinand et al
Phase	Phase III	Phase II/III	Phase III	Phase III	Phase III	Phase II
Randomization	Randomized	Randomized	Randomized	Randomized	Randomized	Randomized
Blinding	Blinding	Double-blind	Double-blind	Double-blind	Double-blind	Double-blind
Primary Endpoint	A1c	A1c	A1c	A1c	A1c	24-h SBP
Study Treatment Period	52 weeks	24 months	24 weeks	24 weeks	52 weeks	26 weeks
Last scheduled visit with PBO	26 weeks	6 months	24 weeks	24 weeks	52 weeks (no PBO)	26 weeks
Background therapy (Add-ons)	Met + TZD	Met mono	SU mono	SGLT2i with or without metformin	Met mono	Stable OAM
Key inclusion/ exclusion criteria						
Age	≥ 18 years	18–75 years	≥ 18 years	≥ 18 years	≥ 18 years	≥ 18 years
T2D duration	NA	≥ 6 months	NA	NA	for ≥ 6 months	NA
A1c	7.0–11.0	7.0–9.5	7.5–9.5	7.0–9.5	7.5–11	7–9.5
BMI	23–45	25–40	≤ 45	≤ 45	≥ 25	NA
Medication	Stable OAM	Diet & exercise / metformin and/or other OAM	Stable SU	SGLT2i with or without metformin for ≥ 3 months	Stable metformin for ≥ 3 months	OAM

*BMI* body mass index, *NA* not applicable for the study’s design, *Met* metformin, *mono* monotherapy, *OAM* oral antihyperglycemic medication, *PBO* placebo, *SBP* systolic blood pressure, *SGLT2i* sodium-glucose cotransporter-2 inhibitors, *SU* sulfonylurea, *T2D* type 2 diabetes, *TZD* thiazolidinediones

### Statistical analysis

The primary objective included mediation analyses of each of the five placebo-controlled trials (Ferdinand et al., AWARD-1, AWARD-5, AWARD-8, and AWARD-10) followed by a meta-analysis pooling the individual mediation results in a random-effect model. In the mediation analyses, the total effect of dulaglutide 1.5 mg vs. Placebo was decomposed into a weight-dependent (i.e., mediated by weight) and weight-independent effect on changes from baseline for SBP, DBP, and pulse pressure. The total effect, weight-dependent effect, and weight-independent effect were estimated via a series of multiple regression models adjusted for covariates including baseline weight, baseline blood pressure, hypertension diagnosis at baseline, and study-specific covariates. To provide more perspective, we also report the estimated “% weight-independent” calculated as the percentage of weight-independent effect with respect to the total effect. A post-hoc sensitivity meta-analysis was completed without AWARD-8 as its background medication differs from other studies. The mediation analyses assumed no other unknown or unmeasured confounding factors besides the adjusted covariates. The computational details of the mediation analysis were provided in Additional file 1: Supplemental Methods.

For the secondary objective, a mediation analysis for dose response was first conducted as described above on AWARD-11 for dulaglutide 4.5 mg vs. dulaglutide 1.5 mg. AWARD-11 also evaluated dulaglutide 3.0 mg vs. dulaglutide 1.5 mg, but these comparisons were not examined in the current report as there was less difference in SBP between dulaglutide 3.0 mg and dulaglutide 1.5 mg at week 26. An adjusted indirect comparison (Bucher method) [21] of dulaglutide 4.5 mg vs. placebo was then conducted using the mediation analysis results from AWARD-11 (4.5 mg vs. 1.5 mg) and AWARD-5 (1.5 mg vs. placebo), as these trials had similar background therapy (Additional file 1: Supplemental Methods). This analysis provided an estimation of the weight-dependent, weight-independent, and total effects of higher dose dulaglutide compared to placebo. A sensitivity analysis of the indirect comparison of dulaglutide 4.5 mg vs. placebo was also conducted that included a subset of participants from Ferdinand et al. which had similar background therapy as AWARD-11 and AWARD-5.

Analyses were based on the intention-to-treat populations from each study, excluding patients who discontinued study drug before 6 months. All analyses were exploratory: descriptive and mediation analysis (PROC CAUSALMED) was performed using SAS v9.4, and the meta-analysis (packages *meta* and *metafor*) and indirect comparison were performed using R v3.4.4.

## Results

### Baseline characteristics and concomitant medications

Baseline characteristics were largely similar across studies (Additional file 1: Table S1). Mean age ranged from 54 to 58 years, percentage of male patients ranged from 44 to 59%, and percentage of patients identified as white ranged from 51 to 89%. Duration of T2D ranged from 6.8 to 9.2 years and baseline SBP ranged from 127 to 132 mmHg. At baseline, 59–72% of participants had a hypertension diagnosis. Concomitant antihyperglycemic and antihypertensive medications at baseline are presented (Additional file 1: Table S2). The majority (88–100%) of participants included in the individual trials received metformin as background therapy, except for participants in AWARD-8, who predominantly received a sulfonylurea due to differences in trial design (Additional file 1: Table S2). Blood pressure, hemoglobin A1c, and body weight results for the dulaglutide 1.5 mg group were comparable for studies used in the indirect comparison analysis for the effect of higher dose dulaglutide (Additional file 1: Table S3).

### SBP change from baseline (dulaglutide 1.5 mg vs. placebo)

**Fig. 2:**
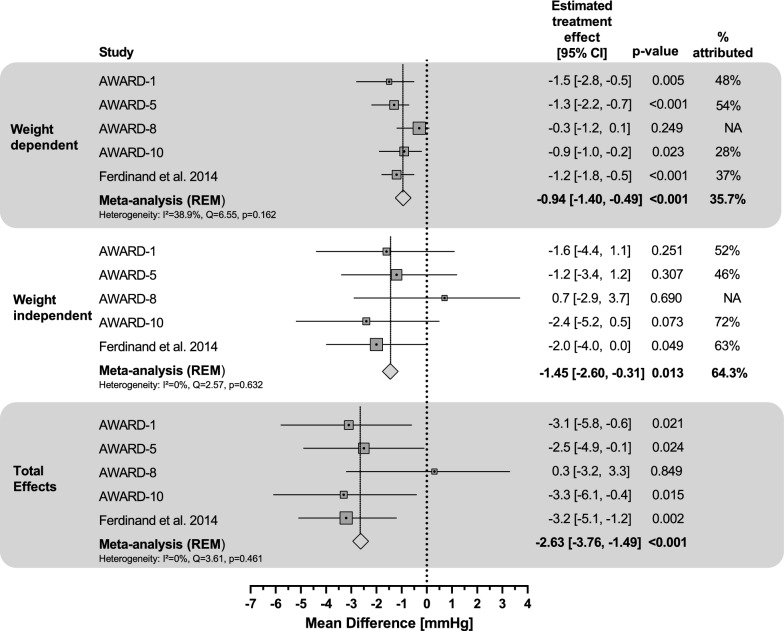
Mediation of dulaglutide 1.5 mg effects on SBP: meta-analysis of placebo-controlled trials for weight dependent vs. weight independent effects. Percent attributed as weight-independent was calculated as (1 − Weight-dependent Effect / Total Effect) × 100% and was reported only when total effect p-value < 0.05 or when the weight-dependent and weight-independent effects had the same sign. *CI* confidence interval, *NA* not applicable, *REM* random-effect model, *SBP* systolic blood pressure

In the mediation meta-analysis of placebo-controlled trials, the estimated overall total effect of dulaglutide 1.5 mg was − 2.6 mmHg (Table 2; Fig. 2), significantly reducing SBP compared to placebo (95% CI: − 3.8, − 1.5; p < 0.001); 36% of dulaglutide’s total effect on BP change was weight-dependent, with an estimated treatment group difference of − 0.9 mmHg (95% CI: − 1.4, − 0.5; p < 0.001). Consequently, the weight-independent effect of dulaglutide 1.5 mg comprised 64% of the total effect, with an estimated treatment effect of − 1.5 mmHg (95% CI: − 2.6, − 0.3; p = 0.013). No significant heterogeneity was detected in the meta-analysis. Results from the post-hoc sensitivity meta-analysis excluding AWARD-8 were consistent with the primary meta-analysis results (Additional file 1; Table S4).

**Table 2:** Summary of findings for SBP and PP on dulaglutide treatment effect in participants with T2D

		Weight-dependent effect (mmHg)	Weight-independent effect (mmHg)	Total effect (mmHg)	% Weight-independent (%)
Mediation meta-analysis of placebo-controlled trials (AWARD-1, 5, 8, 10, 11, and Ferdinand et al.)					
Dula 1.5 mg vs. PBO	SBP	− 0.9 (− 1.4, − 0.5)	− 1.5 (− 2.6, − 0.3)	− 2.6 (− 3.8, − 1.5)	64
	PP	− 0.4 (− 0.6, − 0.1)	− 2.0 (− 3.0, − 1.0)	− 2.5 (− 3.5, − 1.5)	86
Mediation analysis for dose response (AWARD-11)					
Dula 4.5 mg vs. Dula 1.5 mg	SBP	− 0.7 (− 1.1, − 0.4)	− 0.3 (− 1.6, 1.0)	− 1.0 (− 2.2, 0.3)	29
	PP	− 0.4 (− 0.6, − 0.2)	− 0.9 (− 1.8, 0.2)	− 1.2 (− 2.2, − 0.1)	70
Indirect comparison of Dula 4.5 mg vs. PBO (AWARD-5 and 11)					
Dula 4.5 mg vs. PBO	SBP	− 2.0 (− 2.9, − 1.1)	− 1.5 (− 4.2, 1.2)	− 3.5 (− 6.2, − 0.8)	43
	PP	− 1.1 (− 1.7, − 0.5)	− 1.8 (− 4.0, 0.4)	− 2.9 (− 5.1, − 0.7)	62

*Dula* dulaglutide, *PBO* placebo, *PP* pulse pressure, *SBP* systolic blood pressure

% Weight-Independent was calculated as (1 − Weight-dependent Effect / Total Effect) × 100% and was reported only when total effect p-value < 0.05 or when the weight-dependent and weight-independent effects had the same sign

### Pulse pressure change from baseline (dulaglutide 1.5 mg vs. placebo)

**Fig. 3:**
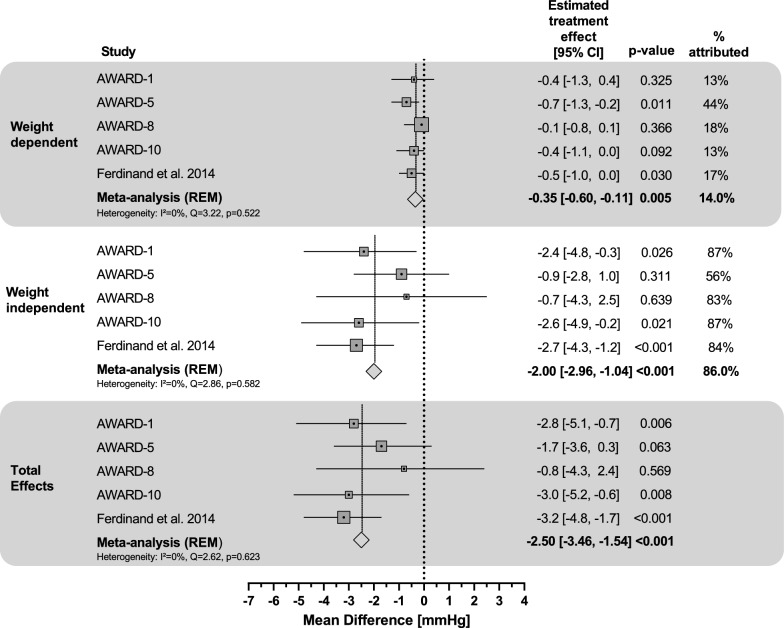
Mediation of dulaglutide 1.5 mg effects on pulse pressure: meta-analysis of placebo-controlled trials for weight dependent vs. weight independent effects. Percent attributed as weight-independent was calculated as (1 − Weight-dependent Effect / Total Effect) × 100% and was reported only when total effect p-value < 0.05 or when the weight-dependent and weight-independent effects had the same sign. *CI* confidence interval, *REM* random-effect model

In the mediation meta-analysis of placebo-controlled trials, the estimated overall total effect of dulaglutide 1.5 mg was − 2.5 mmHg, demonstrating significantly reduced pulse pressure compared to placebo (95% CI: − 3.5, − 1.5; p < 0.001) (Table 2; Fig. 3); 14% of dulaglutide’s total effect on pulse pressure change was weight-dependent, with an estimated overall treatment group difference of − 0.4 mmHg (95% CI: − 0.6, − 0.1; p = 0.005). The weight-independent effect of dulaglutide 1.5 mg comprised 86% of the total effect, with an overall estimated treatment effect of − 2.0 mmHg (95% CI: − 3.0, − 1.0; p < 0.001). The post-hoc sensitivity meta-analysis for pulse pressure showed consistent results (Additional file 1: Table S5).

### DBP change from baseline (dulaglutide 1.5 mg vs. placebo)

In the mediation meta-analysis of placebo-controlled trials, limited effect was seen for DBP. This is likely due to that DBP changes from baseline were generally small in the individual studies (effect estimates ranged from − 0.9 to 1.1 mmHg; all *p* > 0.05 (Additional file 1; Table S6). The overall total effect was minimal and not significant (− 0.2 mmHg; 95% CI: − 1.0, 0.5; p = 0.56); there was a small but significant weight-dependent effect that decreased DBP (− 0.6 mmHg; 95% CI: − 0.8, − 0.3; *p* < 0.001), while the weight-independent effect for increased DBP was not significant (+ 0.5 mmHg; 95% CI: − 0.3, 1.2; p = 0.24). The post-hoc sensitivity meta-analysis for DBP showed consistent results (Additional file 1; Table S6).

### The dose effect of dulaglutide 4.5 mg vs. dulaglutide 1.5 mg and placebo

The mediation analysis for dose response for estimation of difference between dulaglutide 4.5 mg vs. 1.5 mg was conducted on AWARD-11 at week 26. The dose response for SBP estimated total effect for 4.5 mg vs. 1.5 mg was − 1.0 mmHg (95% CI: − 2.2, 0.3; *p* = 0.15), weight-dependent effect was − 0.7 mmHg (95% CI: − 1.1, − 0.4; *p* < 0.001), and the weight-independent effect was − 0.3 mmHg (95% CI: − 1.6, 1.0; *p* = 0.67) (Table 2). For dulaglutide 4.5 mg, 71% of the additional effect beyond dulaglutide 1.5 mg on SBP reduction was weight dependent. When comparing the dose response of dulaglutide 4.5 mg to 1.5 mg at week 26 for pulse pressure, the total effect was − 1.2 mmHg (95% CI: − 2.2, − 0.1; *p* = 0.02), the weight-dependent effect was − 0.4 mmHg (95% CI: − 0.6, − 0.2; *p* = 0.001), and weight-independent effect was − 0.9 mmHg (95% CI: − 1.8, 0.2; *p* = 0.11). For dulaglutide 4.5 mg vs. 1.5 mg, 30% of the effect on pulse pressure reduction was weight-dependent, and 70% of the effect was weight-independent. For dose response between dulaglutide 4.5 mg and 1.5 mg, DBP did not demonstrate a significant total effect (0.3; 95% CI: − 0.6, 1.2; p = 0.56) nor weight-independent effect (0.6; 95% CI: − 0.2, 1.5, p = 0.18) while there was a small weight-dependent effect (− 0.3; 95% CI: − 0.6, − 0.2) p < 0.001) (Additional file 1; Table S7).

The indirect comparison analysis of dulaglutide 4.5 mg vs. placebo using AWARD-11 and AWARD-5 estimated the total effect for SBP change to be − 3.5 mmHg (95% CI: − 6.2, − 0.8; *p* = 0.01; Table 2), the weight-dependent effect to be − 2.0 mmHg (95% CI: − 2.9, − 1.1; *p* < 0.001), and the weight-independent effect to be − 1.5 mmHg (95% CI: − 4.2, 1.2; *p* = 0.28). Of the total effect of dulaglutide 4.5 mg vs. placebo, 57% was weight dependent, and 43% was weight independent. Results of the sensitivity analysis for SBP from the indirect comparison for dulaglutide 4.5 mg vs. placebo, which included the additional data from Ferdinand et al., were consistent with the primary analysis (Additional file 1; Table S8).

Additionally, the indirect comparison of the effect of dulaglutide 4.5 mg vs. placebo estimated the total effect for pulse pressure to be − 2.9 mmHg (95% CI: − 5.1, − 0.7; *p* = 0.01; Tables 2 and S8). The weight-dependent effect was − 1.1 mmHg (95% CI: − 1.7, − 0.5; *p* < 0.001), and the weight-independent effect − 1.8 mmHg (95% CI: − 4.0, 0.4; *p* = 0.11). A total of 38% of the effect was weight-dependent, and 62% of the effect was weight independent. In the sensitivity analysis for the indirect comparison of dulaglutide 4.5 mg vs. placebo there was modest variation from the primary analysis as the total effect was an additional 0.5 mmHg reduction in pulse pressure which was due to a larger weight-independent effect. While the absolute weight-dependent effect was increased moderately compared to the primary analysis, it explained only 29% of the reduction in pulse pressure, and 71% of the reduction was weight-independent (Additional file 1; Table S9).

The indirect comparison for the effect of dulaglutide 4.5 mg vs. placebo for DBP was in-line with the results for dulaglutide 1.5 mg vs. placebo; the estimated total effect was − 0.6 mmHg (95% CI: − 2.2, 1.0; p = 0.46), the weight-dependent effect was -0.9 mmHg (95% CI: − 1.3, − 0.5; p < 0.001), while the weight-independent effect was 0.3 (− 1.3, 1.9), p = 0.71) (Additional file 1; Table S7). The sensitivity analysis for the indirect comparison demonstrated similar results for DBP (Additional file 1; Table S7).

## Discussion

Our primary findings demonstrate that dulaglutide treatment has both weight-dependent and weight-independent effects on reduction in SBP and pulse pressure in participants with T2D. Both SBP and pulse pressure decreased consistently with dulaglutide treatment, and the majority of the effect on blood pressure with dulaglutide 1.5 mg treatment was weight-independent, as weight reduction mediated only 36% and 14% of the effect on SBP and pulse pressure, respectively. Additional reductions of SBP and pulse pressure were observed with dulaglutide 4.5 mg. The greater effect was mostly weight-dependent and was likely driven by the greater weight reduction known to occur with higher dose dulaglutide [22].

### Possible mechanisms of dulaglutide’s effects on CV system

Dulaglutide is a highly efficacious treatment for hyperglycemia in T2D that was developed based on incretin physiology, as GLP-1 is released after nutrient ingestion and stimulates glucose-dependent secretion of insulin. Pleotropic effects of GLP-1 RAs include suppression of glucagon, delayed gastric emptying, and improved satiety [23]. Like other GLP-1 RAs, chronic treatment with dulaglutide is associated with reduced body weight [24], which has been proposed as one of the mechanisms underlying the concomitant decrease in BP. While weight reduction is a favorable characteristic of a T2D treatment, our analysis showed that most of the BP reduction that occurred with dulaglutide 1.5 mg was weight-independent, highlighting additional effects of dulaglutide.

In the REWIND CV outcomes trial evaluating dulaglutide 1.5 mg vs. placebo, 3-point major adverse cardiac events (MACE-3) were reduced by 12% in the dulaglutide arm over a median of 5.4 years, even though 68% of participants at baseline only had CV risk factors and had not experienced a CV event prior to starting the trial [25]. Over the course of the REWIND trial, the estimated treatment difference for SBP was a 1.7-mmHg reduction for dulaglutide compared to placebo [25]. There was also a 1.9-beat-per-minute increase in heart rate with dulaglutide treatment that persisted through the REWIND trial, which was similar to the results of other GLP-1 RA cardiovascular outcomes trials [26]. While the mechanism of the slight increase in heart rate with GLP-1 RA treatment is uncertain, clinical evidence supports that GLP-1 receptor activation does not affect cardiac sympathetic activity [27], and the MACE reduction seen in REWIND and other CVOTs is reassuring. A mediation analysis of the REWIND trial data did not demonstrate a significant relationship between MACE reduction and reduced SBP [28]. A similar analysis of data from the LEADER trial evaluating liraglutide vs. placebo also did not find SBP reduction to be a mediator of MACE reduction [29], however, pulse pressure was not included in either of these mediation analyses.

Increases in pulse pressure are caused by arterial stiffening [30]. Diabetes and obesity are established risk factors for elevated pulse pressure and accelerate the progression of arterial stiffening that occurs with age [30]. Dulaglutide and other GLP-1 RAs improve arterial stiffness in people with T2D [31, 32]. Numerous mechanisms contribute to the arterial stiffness that occurs in patients with obesity, insulin resistance and type 2 diabetes, including endothelial cell and vascular smooth muscle cell dysfunction [33]. Liu et al. reviewed the GLP-1 receptor activation-induced cell signaling that directly improves endothelial cell and vascular smooth muscle dysfunction [34]. Importantly, GLP-1 receptor activation increases endothelial-dependent relaxation and reduces endothelial-induced contractions of vascular smooth muscle to reduce blood pressure [34]. Various other mechanisms have been identified in preclinical models including that GLP-1 receptor agonism directly induces secretion of atrial natriuretic peptide [19]. However, findings are not consistent in clinical studies [35]. More recent research demonstrates that dulaglutide treatment in people with early T2D occurs with increased number and function of circulating endothelial progenitor cells (EPCs), which are important for maintaining endothelial structure by repairing vascular injury. This increase in EPCs predicted a decrease in arterial stiffness supporting the potential clinical relevance on this finding [15]. Other mechanisms proposed for GLP-1 RAs on cardiovascular function and BP reduction include suppression of oxidative stress, anti-inflammatory activity, renal anti-fibrotic effects, and central nervous system control [36–38]. The area postrema, a circumventricular organ located in the dorsal medulla of the brain which densely expresses GLP-1 receptors, may have a role mediating the effects of GLP-1 RAs [39]; preclinical models of hypertension support an antihypertensive effect of GLP-1 RAs by activating these neurons and suppressing sympathetic nerve activity [40].

### Integration of our findings with previous studies

In the current study, data from similar time points (24–36 weeks) were used to limit potential confounding by the length of GLP-1 RA treatment on the mediation analysis results. Another meta-analysis found that weight reduction partially mediated SBP reduction with GLP-1 RA treatment [16]; however, the effect may have been confounded by the broad range of timing used in the study (8 weeks to > 5 years). A third meta-analysis with an endpoint time range of 12–56 weeks did not find an effect of weight reduction on SBP [19]. After 4 weeks, treatment with once weekly dulaglutide contributes to robust decreases in SBP [20, 22] and pulse pressure [20], but partial attrition occurs by week 26. Due to the partial attrition of the BP effect from 4 to 26 weeks and that at 4 weeks the BP reductions are before significant weight reduction, at least a portion of this early decrease in BP is likely due to a different mechanism than the reductions seen at the later time points investigated in the current study.

### Possible explanations for different results in AWARD-8

AWARD-8 was the only individual study included in the primary analysis that did not show a significant effect of treatment for either SBP or pulse pressure; participants in AWARD-8 also had the lowest mean weight reduction of the trials included in this analysis. Differences in the trial design that could account for the different response include the concomitant medications taken by the participants; while participants in AWARD-8 were taking a sulfonylurea, with very few participants receiving concomitant metformin, participants in the other trials were predominantly on metformin with or without another oral anti-diabetes medication. In a retrospective cohort comparing monotherapy after one year of treatment with sulfonylurea, SBP was 1.3 mmHg higher vs. metformin, and this difference was thought to be due to the varying treatment effects on weight [41]. The literature regarding the effect of metformin on BP suggests there may be a small BP-lowering effect that occurs specifically in individuals with underlying hypertension [42]. Even with potentially different effects on BP between sulfonylurea and metformin, participants were required to be on stable background therapy before enrolling in the included trials. AWARD-8 also had the smallest sample size for a single study which limits any generalizability to the findings from this individual study.

### Active weight loss vs. weight reduction maintenance

While there is a robust decrease in BP during active weight loss, the benefit is attenuated during the weight reduction maintenance phase. This attenuation is hypothesized to be due to a lack of sustained neurohormonal responses to the active weight loss during weight reduction maintenance [17]. In the Look AHEAD trial, participants with T2D who undertook an intensive lifestyle-based weight-reduction intervention achieved an 8.6% weight reduction by the end of one year compared to 0.7% in the standard diabetes education arm, concurrent with a 7-mmHg decrease in SBP [43]. After approximately 10 years of follow-up, most of the weight reduction (6.0%) was maintained in the intensive arm but only 2 mmHg of the SBP reduction persisted [44]. Pulse pressure also decreases early on after lifestyle-induced weight reduction phase in people with obesity [45]. While long-term follow up from bariatric surgery also demonstrates a reversal of the early reduction in SBP and DBP, there is long-term slowing of the age-associated increase in pulse pressure [46]. In the REWIND trial, after a median of 5.4 years of follow up the dulaglutide treatment occurred with associated reductions in SBP (− 1.7 mmHg) and weight (− 1.5 kg) [25], but it is not known if weight reduction contributes to the long-term SBP effect observed in the REWIND trial.

### Weight-independent and weight-dependent effects of dulaglutide on blood pressure

The Ferdinand et al. 2014 trial included in this meta-analysis reported a differential dose effect of dulaglutide 0.75 mg vs. 1.5 mg on SBP and pulse pressure [19]. In the current study, we also found a dose effect for SBP and pulse pressure reduction when comparing dulaglutide 4.5 mg and 1.5 mg at 26 weeks in AWARD-11. The higher dose reduced SBP by an additional − 1.0 mmHg compared to lower dose of which 71% was dependent on weight reduction and reduced pulse pressure by an additional 1.2 mmHg of which 30% was dependent on weight reduction. Interestingly, the weight-independent effect of dulaglutide 1.5 mg and dulaglutide 4.5 mg were similar (− 1.5 mmHg for SBP and − 2.0 mmHg and − 1.8 mmHg for pulse pressure, respectively), suggesting that the weight-independent effect of dulaglutide may be maximized at the 1.5 mg dose. Thus, that may be the limit of the weight-independent effects of dulaglutide.

### Study limitations

There are specific considerations which limit the interpretation of the current study. Many of the clinical trials included were multinational impacting the racial and ethnic representation; therefore, it is uncertain if the current findings apply to specific populations with high rates of T2D and high blood pressure such as African American or Black adults. Participants’ blood pressure control at baseline was potentially better than many clinical practice populations and may also influence generalizability of the findings. Additionally, because the trials included in this analysis were primarily designed to assess hyperglycemia, strict definitions for hypertension diagnosis at baseline were not used, partially limiting interpretation. Our findings are specific to approximately 6 months of dulaglutide treatment; additional research is necessary to understand the interaction of dulaglutide and weight reduction on SBP and pulse pressure in longer-term studies. Results from the indirect comparison should be interpreted with caution as despite the similarities in baseline demographics and background treatment there might be unmeasured confounding factors that could have influenced the results.

## Conclusions

In conclusion, as elevated SBP and pulse pressure are risk factors for cardiovascular and microvascular complications in patients with T2D, treatment options like dulaglutide and other GLP-1 RAs that reduce these are favorable. The current findings indicate that a portion of the SBP and pulse pressure reduction observed with dulaglutide treatment is not weight mediated, and further research is needed to understand the mechanisms of the additional benefit. Understanding the mechanisms by which dulaglutide improves SBP and pulse pressure, whether dependent or independent of weight reduction, could provide insight into developing future treatment regimens for elevated blood pressure.

## Supplementary Information

**Additional file 1.** Supplemental Methods and Tables S1-S9.

## Data Availability

Eli Lilly and Company provides access to all individual participant data collected during the trial, after anonymization, with the exception of pharmacokinetic or genetic data. Data are available to request 6 months after the indication studied has been approved in the US and EU and after primary publication acceptance, whichever is later. No expiration date of data requests is currently set once data are made available. Access is provided after a proposal has been approved by an independent review committee identified for this purpose and after receipt of a signed data sharing agreement. Data and documents, including the study protocol, statistical analysis plan, clinical study report, blank or annotated case report forms, will be provided in a secure data sharing environment. For details on submitting a request, see the instructions provided at www.vivli.org.

## Abbreviations

BP: Blood pressure
DBP: Diastolic BP
dula: Dulaglutide
GLP-1: Glucagon-like peptide-1
GLP-1 RA: GLP-1 receptor agonist
MACE: Major adverse cardiovascular events
MACE-3: 3-Point major adverse cardiac events
PBO: Placebo
SBP: Systolic BP
T2D: Type 2 diabetes

## References

[1] Muntner P, Whelton PK, Woodward M, Carey RM (2018). A comparison of the 2017 American College of Cardiology/American Heart Association blood pressure guideline and the 2017 American Diabetes Association diabetes and hypertension position statement for U.S. adults with diabetes. Diabetes Care.

[2] An J, Nichols GA, Qian L, Munis MA, Harrison TN, Li Z (2021). Prevalence and incidence of microvascular and macrovascular complications over 15 years among patients with incident type 2 diabetes. BMJ Open Diabetes Res Care.

[3] Tseng LN, Tseng YH, Jiang YD, Chang CH, Chung CH, Lin BJ (2012). Prevalence of hypertension and dyslipidemia and their associations with micro- and macrovascular diseases in patients with diabetes in Taiwan: an analysis of nationwide data for 2000–2009. J Formos Med Assoc.

[4] Flack JM, Adekola B (2020). Blood pressure and the new ACC/AHA hypertension guidelines. Trends Cardiovasc Med.

[5] Kannel WB (1995). Framingham study insights into hypertensive risk of cardiovascular disease. Hypertens Res.

[6] Whelton PK, He J, Appel LJ, Cutler JA, Havas S, Kotchen TA (2002). Primary prevention of hypertension: clinical and public health advisory from The National High Blood Pressure Education Program. JAMA.

[7] Blood Pressure Lowering Treatment Trialists’ Collaboration (2021). Pharmacological blood pressure lowering for primary and secondary prevention of cardiovascular disease across different levels of blood pressure: an individual participant-level data meta-analysis. Lancet.

[8] Reboldi G, Gentile G, Angeli F, Ambrosio G, Mancia G, Verdecchia P (2011). Effects of intensive blood pressure reduction on myocardial infarction and stroke in diabetes: a meta-analysis in 73,913 patients. J Hypertens.

[9] Adler AI, Stratton IM, Neil HA, Yudkin JS, Matthews DR, Cull CA (2000). Association of systolic blood pressure with macrovascular and microvascular complications of type 2 diabetes (UKPDS 36): prospective observational study. BMJ.

[10] Whelton PK, Carey RM, Aronow WS, Casey DE, Collins KJ, Dennison Himmelfarb C (2018). 2017 ACC/AHA/AAPA/ABC/ACPM/AGS/APhA/ASH/ASPC/NMA/PCNA guideline for the prevention, detection, evaluation, and management of high blood pressure in adults: a report of the American College of Cardiology/American Heart Association Task Force on Clinical Practice Guidelines. Hypertension.

[11] Ali MK, Bullard KM, Saaddine JB, Cowie CC, Imperatore G, Gregg EW (2013). Achievement of goals in U.S. diabetes care, 1999–2010. N Engl J Med.

[12] Grenier J, Goodman SG, Leiter LA, Langer A, Teoh H, Bhatt DL (2018). Blood pressure management in adults with type 2 diabetes: insights from the diabetes mellitus status in Canada (DM-SCAN) survey. Can J Diabetes.

[13] Berra C, Manfrini R, Regazzoli D, Radaelli MG, Disoteo O, Sommese C (2020). Blood pressure control in type 2 diabetes mellitus with arterial hypertension. The important ancillary role of SGLT2-inhibitors and GLP1-receptor agonists. Pharmacol Res.

[14] Palmer SC, Tendal B, Mustafa RA, Vandvik PO, Li S, Hao Q (2021). Sodium-glucose cotransporter protein-2 (SGLT-2) inhibitors and glucagon-like peptide-1 (GLP-1) receptor agonists for type 2 diabetes: systematic review and network meta-analysis of randomised controlled trials. BMJ.

[15] Xie D, Li Y, Xu M, Zhao X, Chen M (2022). Effects of dulaglutide on endothelial progenitor cells and arterial elasticity in patients with type 2 diabetes mellitus. Cardiovasc Diabetol.

[16] Ryan D, Acosta A (2015). GLP-1 receptor agonists: nonglycemic clinical effects in weight loss and beyond. Obesity (Silver Spring).

[17] Hall ME, Cohen JB, Ard JD, Egan BM, Hall JE, Lavie CJ (2021). Weight-loss strategies for prevention and treatment of hypertension: a scientific statement from the American Heart Association. Hypertension.

[18] Hu M, Cai X, Yang W, Zhang S, Nie L, Ji L (2020). Effect of hemoglobin a1c reduction or weight reduction on blood pressure in glucagon-like peptide-1 receptor agonist and sodium-glucose cotransporter-2 inhibitor treatment in type 2 diabetes mellitus: a meta-analysis. J Am Heart Assoc.

[19] Katout M, Zhu H, Rutsky J, Shah P, Brook RD, Zhong J (2013). Effect of GLP-1 mimetics on blood pressure and relationship to weight loss and glycemia lowering: results of a systematic meta-analysis and meta-regression. Am J Hypertens.

[20] Ferdinand KC, White WB, Calhoun DA, Lonn EM, Sager PT, Brunelle R (2014). Effects of the once-weekly glucagon-like peptide-1 receptor agonist dulaglutide on ambulatory blood pressure and heart rate in patients with type 2 diabetes mellitus. Hypertension.

[21] Bucher HC, Guyatt GH, Griffith LE, Walter SD (1997). The results of direct and indirect treatment comparisons in meta-analysis of randomized controlled trials. J Clin Epidemiol.

[22] Frias JP, Bonora E, Nevarez Ruiz L, Li YG, Yu Z, Milicevic Z (2021). Efficacy and safety of dulaglutide 3.0 mg and 4.5 mg versus dulaglutide 1.5 mg in metformin-treated patients with type 2 diabetes in a randomized controlled trial (AWARD-11). Diabetes Care.

[23] Jendle J, Grunberger G, Blevins T, Giorgino F, Hietpas RT, Botros FT (2016). Efficacy and safety of dulaglutide in the treatment of type 2 diabetes: a comprehensive review of the dulaglutide clinical data focusing on the AWARD phase 3 clinical trial program. Diabetes Metab Res Rev.

[24] Bonora E, Frias JP, Tinahones FJ, Van J, Malik RE, Yu Z (2021). Effect of dulaglutide 3.0 and 4.5 mg on weight in patients with type 2 diabetes: exploratory analyses of AWARD-11. Diabetes Obes Metab.

[25] Gerstein HC, Colhoun HM, Dagenais GR, Diaz R, Lakshmanan M, Pais P (2019). Dulaglutide and cardiovascular outcomes in type 2 diabetes (REWIND): a double-blind, randomised placebo-controlled trial. Lancet.

[26] Das SR, Everett BM, Birtcher KK, Brown JM, Januzzi JL, Kalyani RR (2020). 2020 Expert consensus decision pathway on novel therapies for cardiovascular risk reduction in patients with type 2 diabetes: a report of the American College of Cardiology Solution Set Oversight Committee. J Am Coll Cardiol.

[27] Bharucha AE, Charkoudian N, Andrews CN, Camilleri M, Sletten D, Zinsmeister AR (2008). Effects of glucagon-like peptide-1, yohimbine, and nitrergic modulation on sympathetic and parasympathetic activity in humans. Am J Physiol Regul Integr Comp Physiol.

[28] Konig M, Riddle MC, Colhoun HM, Branch KR, Atisso CM, Lakshmanan MC (2021). Exploring potential mediators of the cardiovascular benefit of dulaglutide in type 2 diabetes patients in REWIND. Cardiovasc Diabetol.

[29] Buse JB, Bain SC, Mann JFE, Nauck MA, Nissen SE, Pocock S (2020). Cardiovascular risk reduction with liraglutide: an exploratory mediation analysis of the LEADER trial. Diabetes Care.

[30] Schillaci G, Pucci G (2015). Aging and pulse pressure widening: the inseparable duo?. J Hypertens.

[31] Lambadiari V, Pavlidis G, Kousathana F, Varoudi M, Vlastos D, Maratou E (2018). Effects of 6-month treatment with the glucagon like peptide-1 analogue liraglutide on arterial stiffness, left ventricular myocardial deformation and oxidative stress in subjects with newly diagnosed type 2 diabetes. Cardiovasc Diabetol.

[32] Tuttolomondo A, Cirrincione A, Casuccio A, Del Cuore A, Daidone M, Di Chiara T (2021). Efficacy of dulaglutide on vascular health indexes in subjects with type 2 diabetes: a randomized trial. Cardiovasc Diabetol.

[33] Jia G, Aroor AR, DeMarco VG, Martinez-Lemus LA, Meininger GA, Sowers JR (2015). Vascular stiffness in insulin resistance and obesity. Front Physiol.

[34] Liu L, Liu J, Huang Y (2015). Protective effects of glucagon-like peptide 1 on endothelial function in hypertension. J Cardiovasc Pharmacol.

[35] Lovshin JA, Barnie A, DeAlmeida A, Logan A, Zinman B, Drucker DJ (2015). Liraglutide promotes natriuresis but does not increase circulating levels of atrial natriuretic peptide in hypertensive subjects with type 2 diabetes. Diabetes Care.

[36] Yaribeygi H, Farrokhi FR, Abdalla MA, Sathyapalan T, Banach M, Jamialahmadi T (2021). The effects of glucagon-like peptide-1 receptor agonists and dipeptydilpeptidase-4 inhibitors on blood pressure and cardiovascular complications in diabetes. J Diabetes Res.

[37] Helmstadter J, Frenis K, Filippou K, Grill A, Dib M, Kalinovic S (2020). Endothelial GLP-1 (glucagon-like peptide-1) receptor mediates cardiovascular protection by liraglutide in mice with experimental arterial hypertension. Arterioscler Thromb Vasc Biol.

[38] Mohamad HE, Abdelhady MA, Abdel Aal SM, Elrashidy RA (2022). Dulaglutide mitigates high dietary fructose-induced renal fibrosis in rats through suppressing epithelial-mesenchymal transition mediated by GSK-3β/TGF-β1/Smad3 signaling pathways. Life Sci.

[39] Yamamoto H, Kishi T, Lee CE, Choi BJ, Fang H, Hollenberg AN (2003). Glucagon-like peptide-1-responsive catecholamine neurons in the area postrema link peripheral glucagon-like peptide-1 with central autonomic control sites. J Neurosci.

[40] Katsurada K, Nakata M, Saito T, Zhang B, Maejima Y, Nandi SS (2019). Central glucagon-like peptide-1 receptor signaling via brainstem catecholamine neurons counteracts hypertension in spontaneously hypertensive rats. Sci Rep.

[41] Roumie CL, Liu X, Choma NN, Greevy RA, Hung AM, Grijalva CG (2012). Initiation of sulfonylureas versus metformin is associated with higher blood pressure at one year. Pharmacoepidemiol Drug Saf.

[42] Anabtawi A, Miles JM (2016). Metformin: nonglycemic effects and potential novel indications. Endocr Pract.

[43] Look ARG, Pi-Sunyer X, Blackburn G, Brancati FL, Bray GA, Bright R (2007). Reduction in weight and cardiovascular disease risk factors in individuals with type 2 diabetes: one-year results of the look AHEAD trial. Diabetes Care.

[44] Wing RR, Bolin P, Brancati FL, Bray GA, Clark JM, Coday M (2013). Cardiovascular effects of intensive lifestyle intervention in type 2 diabetes. N Engl J Med.

[45] Kwagyan J, Tabe CE, Xu S, Maqbool AR, Gordeuk VR, Randall OS (2005). The impact of body mass index on pulse pressure in obesity. J Hypertens.

[46] Sjöström CD, Peltonen M, Sjöström L (2001). Blood pressure and pulse pressure during long-term weight loss in the obese: the Swedish Obese Subjects (SOS) Intervention Study. Obes Res.

