# Supplementary material for: Weight-dependent and weight-independent effects of dulaglutide on blood pressure in patients with type 2 diabetes
Source: Cardiovasc Diabetol. 2023 Mar 9;22:49. doi: 10.1186/s12933-023-01775-x (PMC9999488; doi:10.1186/s12933-023-01775-x)
Supplement: Supplementary file 1 — Additional file 1. Supplemental Methods and Tables S1-S9. [file 12933_2023_1775_MOESM1_ESM.docx]

**Supplemental Methods**

*Mediation analysis*

Mediation analysis was conducted in each of the five placebo-controlled trials respectively (Ferdinand et al., AWARD-1, AWARD-5, AWARD-8, and AWARD-10). In each of the mediation analysis, the total effect of dulaglutide vs placebo on blood pressure changes (BP) can be statistically decomposed into an indirect “mediation” effect and a “direct” effect. The mediation effect is the effect of dulaglutide on blood pressure changes attributable to the weight (WT) reduction (mediator) caused by dulaglutide treatment. The direct effect is the dulaglutide effect adjusted for weight reduction. In our context, mediation effect is the “weight-dependent” dulaglutide effect, and the direct effect is the “weight-independent” effect. The implementation of the analysis included four linear regressions (Figure 1).

1. Total-Effect regression: BP change = dulaglutide + covariates, this regression is used to assess the total effect of dulaglutide on BP change

2. Direct-Effect regression: BP change = dulaglutide + weight change + covariates, this regression is used to assess the direct effect of dulaglutide on BP change

3. Mediator-Effect regression: Weight change = dulaglutide + covariates, this regression is used to assess the mediator effect on BP change

4. Linking-Effect regression: BP change = weight change + covariates, this regression is used to assess the linking effect of weight change on BP change

We denoted the mediator coefficient as the coefficient of dulaglutide from regression 3, and the linking coefficient as the coefficient of weight change in regression 4. The weight-dependent effect (mediation effect) is calculated as (mediator coefficient x linking coefficient). The total effect is the regression coefficient of dulaglutide in regression 1. The weight-independent effect (direct effect) is the regression coefficient of dulaglutide in regression 2. The weight-dependent effect can also be calculated as total effect – weight-independent effect in our case when both the outcome and mediator are continuous variables. The parameter of “percent mediated” is defined as weight-dependent effect divided by total effect and was reported when total effect p-value < 0.05 or when the weight-dependent and weight-independent effects had the same sign. The parameter for “percent weight-independent” is calculated as 100% minus “percent mediated”.

All regressions were adjusted for covariates including baseline weight, baseline BP, hypertension diagnosis at baseline, and other study specific covariates listed in the table below. Bootstrapped standard errors of the effect estimates obtained from mediation analysis were used to derive 95% confidence intervals and p-values.

A similar mediation analysis was conducted in AWARD-11 for dulaglutide 4.5 mg vs 1.5 mg.

**Study-specific covariates:**

| **Study Code** | **Covariates** |
| --- | --- |
| AWARD-1 | Country |
| AWARD-5 | Country |
| AWARD-8 | Pooled Country  Baseline A1c stratum < 8.5% (Yes, No) |
| AWARD-10 | Country  Baseline SGLT dose (High, Low)  Baseline metformin use (Yes, No) |
| AWARD-11 | Pooled Country  Baseline A1c stratum < 8.5% (Yes, No) |
| Ferdinand et al. | Pooled Investigator  Baseline hypertension (Yes, No) |


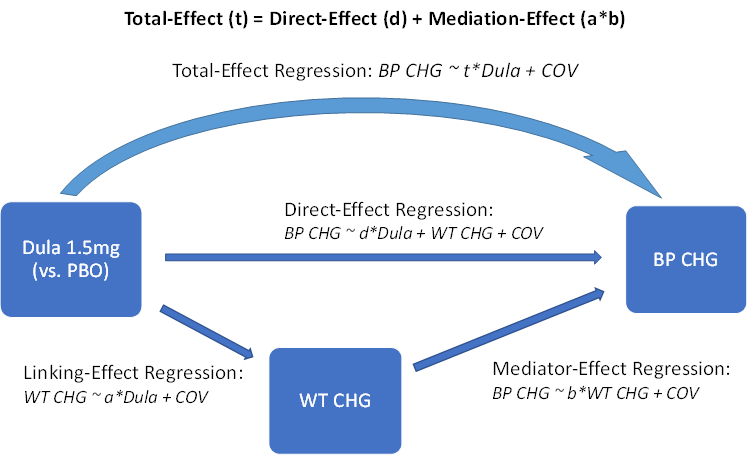


Abbreviations: BP, blood pressure; CHG, change; COV, covariates; Dula, dulaglutide; PBO, placebo; WT, weight.

*Meta-Analysis*

The effect estimates and associated standard errors for the total, weight-dependent and weight-independent effects derived from the study-specific mediation analyses described above were then combined across studies via a random-effects meta-analysis (inverse variance weighting), respectively. Heterogeneity was evaluated via the *I²* statistic and the Q test.

A post-hoc sensitivity meta-analysis was completed without AWARD-8 as its background medication differs from other studies.

*Indirect Comparison*

An indirect comparison of the total effect, weight-dependent effect, and weight-independent effect of dulaglutide high dose (4.5 mg) vs placebo for blood pressure and pulse pressure change was performed using mediation analyses results from AWARD-11 (4.5 mg vs 1.5 mg) and AWARD-5 (1.5 mg vs placebo). Bucher’s method was used for indirect comparison: the effect (or variance) estimate of the high-dose vs placebo was calculated as the sum of the individual effect (or variance) estimates from AWARD-11 and AWARD-5. Confidence intervals and p-values were derived based on Normal approximation using the effect and variance estimates (i.e., WALD-test).

Dulaglutide effect estimate (4.5 mg vs placebo) = AWARD-11 Effect Estimate (4.5 mg vs 1.5 mg) + AWARD-5 Effect Estimate (Dula 1.5 mg vs PBO)

Variance of dulaglutide effect estimate (4.5 mg vs placebo) = variance estimate from AWARD-11 + variance estimate from AWARD-5.

A sensitivity analysis of the indirect comparison of dulaglutide 4.5mg vs placebo was also conducted that included a subset of participants from Ferdinand et al. which had similar background therapy as AWARD-11 and AWARD-5. For this analysis, mediation analysis results for the subset of Ferdinand et al. and AWARD-5 were first combined via a random-effects meta-analysis as described above before the indirect comparison with AWARD-11.

*Analysis Software*

The mediation analysis was performed using SAS v9.4 (PROC CAUSALMED), and the meta-analysis (packages *meta* and *metafor*) and indirect comparison were performed using R v3.4.4.

**Table S1. Baseline characteristics**

|  | Ferdinand et al. | | AWARD-1 | | AWARD-5 | | AWARD-8 | | AWARD-10 | | AWARD-11 | |
| --- | --- | --- | --- | --- | --- | --- | --- | --- | --- | --- | --- | --- |
| Parameters | Dula 1.5 mg  (N=251) | PBO  (N=250) | Dula 1.5 mg  (N=279) | PBO  (N=141) | Dula 1.5 mg  (N=304) | PBO  (N=177) | Dula 1.5 mg  (N=239) | PBO  (N=60) | Dula 1.5 mg  (N=142) | PBO  (N=140) | Dula 4.5 mg  (N=614) | Dula 1.5 mg  (N=612) |
| Age (years),  mean (SD) | 56.0 (10.1) | 56.4 (10.5) | 56.2 (9.7) | 54.6 (10.0) | 53.7 (10.0) | 54.9 (9.0) | 57.7 (10.2) | 58.2 (7.4) | 56.2 (9.3) | 57.1 (9.6) | 56.6 (10.2) | 57.8 (9.7) |
| Male sex, n (%) | 130 (51.8) | 131 (52.4) | 163 (58.4) | 83 (58.9) | 146 (48.0) | 90 (50.8) | 104 (43.5) | 28 (46.7) | 77 (54.2) | 66 (47.1) | 318 (51.8) | 298 (48.7) |
| Race, n (%)  White  Asian  Black  Other | 201 (80.1)  22 (8.8)  23 (9.2)  5 (2.0) | 203 (81.2)  23 (9.2)  22 (8.8)  2 (0.8) | 205 (73.5)  6 (2.2)  24 (8.6)  44 (15.8) | 103 (73.0)  6 (4.3)  10 (7.1)  22 (15.6) | 157 (51.6)  77 (25.3)  16 (5.3)  54 (17.8) | 91 (51.4)  39 (22.0)  9 (5.1)  38 (21.5) | 202 (84.5)  3 (1.3)  7 (2.9)  27 (11.3) | 47 (78.3)  2 (3.3)  4 (6.7)  7 (11.6) | 127 (89.4)  0 (0.0)  3 (2.1)  12 (8.4) | 124 (88.6)  0 (0.0)  6 (4.3)  10 (7.1) | 530 (86.3)  14 (2.3)  23 (3.7)  47 (7.7) | 529 (86.4)  13 (2.1)  28 (4.6)  42 (6.9) |
| Ethnicity  Hispanic or Latino, n (%) | 95 (37.8) | 91 (36.4) | 93 (33.0) | 45 (31.9) | 54 (17.8) | 38 (21.5) | 112 (46.9) | 27 (45.0) | 51 (36.0) | 44 (31.4) | 213 (34.7) | 214 (35.0) |
| T2D duration (years)  mean (SD) | 7.61 (5.3) | 8.4 (5.8) | 8.76 (5.59) | 8.60 (5.8) | 6.95 (5.5) | 7.0 (5.4) | 7.8 (5.3) | 6.8 (3.8) | 9.2 (5.7) | 8.9 (6.1) | 7.7 (5.8) | 7.6 (5.8) |
| Hypertension | 168 (66.9) | 166 (66.4) | 191 (68.5) | 95 (67.4) | 214 (70.4) | 126 (71.2) | 142 (59.4) | 34 (56.7) | NA | NA | 405 (66.0) | 442 (72.2) |
| A1c (%)  mean (SD) | 7.9 (0.8) | 7.9 (0.8) | 8.1 (1.3) | 8.1 (1.3) | 8.1 (1.1) | 8.1 (1.1) | 8.4 (0.7) | 8.4 (0.7) | 8.0 (0.6) | 8.0 (0.7) | 8.6 (0.9) | 8.6 (0.9) |
| BMI, mean (SD) | 32.8 (5.7) | 33.5 (6.5) | 33.1 (5.3) | 32.9 (5.7) | 31.4 (4.6) | 31.4 (4.2) | 30.9 (5.2) | 32.4 (5.9) | 32.9 (5.6) | 32.4 (5.0) | 34.0 (6.2) | 34.4 (6.4) |
| SBP (mmHg)  mean (SD) | 127.3 (10.4) | 127.6 (9.7) | 131.8 (15.3) | 130.1 (15.6) | 128.6 (12.8) | 128.2 (13.4) | 131.5 (13.4) | 130.2 (11.9) | 129.7 (14.5) | 130.6 (13.7) | 132.1 (14.0) | 132.1 (14.2) |
| PP (mmHg) mean (SD) | 50.8 (11.1) | 50.7 (10.8) | 49.8 (12.4) | 48.7 (11.3) | 50.7 (10.9) | 50.5 (11.2) | 53.2 (11.3) | 52.0 (10.2) | 52.6 (12.5) | 52.2 (12.0) | 53.3 (11.3) | 53.1 (11.3) |
| EGFR (ml/min)  mean (SD) | 89.6 (18.1) | 88.8 (17.8) | NC | NC | NC | NC | 90.2 (18.3) | 88.0 (17.4) | 90.9 (18.8) | 90.9 (16.9) | 93.7 (18.3) | 93.4 (18.2) |
| CrCl (ml/min), mean (SD) | NC | NC | 121.2 (40.1) | 121.0 (38.9) | 120.8 (34.6) | 115.9 (29.7) | NC | NC | NC | NC | NC | NC |

BMI, body mass index; CrCl, creatinine clearance; dula, dulaglutide; EGFR, estimated glomerular filtration rate; NC, not collected; PBO, placebo; PP, pulse pressure; SD, standard deviation; SBP, systolic blood pressure; T2D, type 2 diabetes.

**Table S2. Antihyperglycemic medications received at baseline by study**

|  | Ferdinand et al. | | AWARD-1 | | AWARD-5 | | AWARD-8* | | AWARD-10 | | AWARD-11 | |
| --- | --- | --- | --- | --- | --- | --- | --- | --- | --- | --- | --- | --- |
|  | Dula  1.5 mg  (n=251) | PBO  (n=250) | Dula  1.5 mg  (n=279) | PBO  (n=141) | Dula  1.5 mg  (n=304) | PBO  (n=177) | Dula  1.5 mg  (n=239) | PBO  (n=60) | Dula  1.5 mg  (n=142) | PBO  (n=140) | Dula  1.5 mg  (n=612) | Dula  4.5 mg  (n=614) |
| Taking antihyperglycemic medication, n (%) | 251 (100.0) | 250 (100.0) | 273 (97.8) | 139 (98.6) | 290 (95.4) | 167 (94.4) | 239 (100.0) | 60 (100.0) | 142 (100.0) | 140 (100.0) | 612 (100.0) | 614 (100.0) |
| Metformin, n (%) | 229 (91.2) | 233 (93.2) | 255 (91.4) | 133 (94.3) | 268 (88.2) | 156 (88.1) | 15 (6.3) | 7 (11.7) | 136 (95.8) | 135 (96.4) | 612 (100.0) | 614 (100.0) |
| Sulfonylureas, n (%) | 153 (61.0) | 153 (61.2) | 190 (68.1) | 79 (56.0) | 96 (31.6) | 48 (27.1) | 239 (100.0) | 60 (100.0) | 0 | 0 | 0 | 0 |
| Thiazolidinediones, n (%) | 27 (10.8) | 31 (12.4) | 71 (25.4) | 38 (27.0) | 9 (3.0) | 8 (4.5) | 0 | 0 | 0 | 0 | 0 | 0 |
| SGLT2i, n (%) | 0 | 0 | 0 | 0 | ≤1** (0.3) | ≤4** (2.3) | ≤1** (0.4) | 0 | 142 (100.0) | 140 (100.0) | 0 | 0 |
| Antihypertensives, n (%) | 175 (69.7) | 178 (71.2) | 206 (73.8) | 102 (72.3) | 175 (57.6) | 108 (61.0) | 158 (66.1) | 41 (68.3) | 97 (68.3) | 99 (70.7) | 456 (74.5) | 417 (67.9) |
| ACE inhibitors, n (%) | 109 (62.3) | 110 (61.8) | 128 (45.9) | 68 (48.2) | 99 (32.6) | 57 (32.2) | 96 (40.2) | 23 (38.3) | 59 (41.5) | 52 (37.1) | 262 (42.8) | 237 (38.6) |
| Diuretics, n (%) | 59 (33.7) | 49 (27.5) | 62 (22.2) | 35 (24.8) | 67 (22.0) | 50 (28.2) | 83 (34.7) | 21 (35.0) | 31 (21.8) | 37 (26.4) | 182 (29.7) | 171 (27.9) |
| Angiotensin Receptor  Blockers, n (%) | 42 (24.0) | 51 (28.7) | 56 (20.1) | 24 (17.0) | 54 (17.8) | 36 (20.3) | 48 (20.1) | 13 (21.7) | 35 (24.6) | 40 (28.6) | 140 (22.9) | 132 (21.5) |
| Beta blockers, n (%) | 41 (23.4) | 35 (19.7) | 55 (19.7) | 25 (17.7) | 46 (15.1) | 33 (18.6) | 64 (26.8) | 12 (20.0) | 36 (25.4) | 41 (29.3) | 193 (31.5) | 159 (25.9) |
| Calcium channel blockers,  n (%) | 39 (22.3) | 34 (19.1) | 41 (14.7) | 17 (12.1) | 42 (13.8) | 27 (15.3) | 37 (15.5) | 13 (21.7) | 26 (18.3) | 33 (23.6) | 124 (20.3) | 110 (17.9) |
| Antiadrenergic agents, n (%) | 7 (4.0) | 5 (2.8) | 4 (1.4) | 2 (1.4) | 5 (1.6) | 5 (2.8) | 4 (1.7) | 2 (3.3) | 8 (5.6) | 6 (4.3) | 15 (2.5) | 23 (3.7) |
| Renin inhibitors, n (%) | 0 (0.0) | 2 (1.1) | 2 (0.7) | 0 (0.0) | 3 (1.0) | 1 (0.6) | 0 (0.0) | 0 (0.0) | 0 (0.0) | 0 (0.0) | 0 (0.0) | 1 (0.2) |
| Other, n (%) | 0 (0.0) | 0 (0.0) | 9 (3.2) | 3 (2.1) | 1 (0.3) | 4 (2.3) | 0 (0.0) | 0 (0.0) | 1 (0.7) | 3 (2.1) | 2 (0.3) | 1 (0.2) |

*Baseline data was not available; numbers include medications prior to and during treatment period.

**Uncoded medication.

Dula, dulaglutide; PBO, placebo; SGLT2i, sodium-glucose cotransporter-2 inhibitors.

**Table S3. Blood pressure, hemoglobin A1c, and body weight results for the common reference group dulaglutide 1.5 mg in studies used for indirect comparison of dulaglutide 4.5mg vs placebo and associated sensitivity analysis**

| Parameter | AWARD-11  Dulaglutide 1.5 mg | AWARD-5  Dulaglutide 1.5 mg | Ferdinand et al. subset  Dulaglutide 1.5 mg |
| --- | --- | --- | --- |
|  |  |  |  |
| SBP |  |  |  |
| Baseline |  |  |  |
| n | 612 | 304 | 84 |
| Observed mean (SD) | 132.1 (14.2) | 128.6 (12.8) | 124.0 (12.4) |
| Month 6 |  |  |  |
| n | 574 | 271 | 67 |
| Observed mean (SD) | 130.6 (14.0) | 126.4 (12.6) | 121.5 (13.3) |
| LS mean (SE) | 130.3 (0.5) | NA | NA |
| Change from Baseline |  |  |  |
| Observed mean change (SD) | -1.7 (12.7) | -2.4 (11.3) | -1.9 (11.2) |
| LS mean change (SE) | -1.6 (0.5) | -1.7 (0.7) | NA |
| DBP |  |  |  |
| Baseline |  |  |  |
| n | 612 | 304 | 84 |
| Observed mean (SD) | 78.8 (9.3) | 77.9 (8.3) | 75.8 (8.7) |
| Month 6 |  |  |  |
| N | 574 | 271 | 67 |
| Observed mean (SD) | 78.6 (9.2) | 77.7 (8.2) | 77.2 (8.9) |
| LS mean (SE) | 78.6 (0.31) | NA | NA |
| Change from Baseline |  |  |  |
| Observed mean change (SD) | -0.2 (8.5) | -0.7 (7.2) | 1.4 (8.0) |
| LS mean change (SE) | -0.2 (0.3) | -0.4 (0.4) | NA |
| Pulse pressure |  |  |  |
| Baseline |  |  |  |
| n | 612 | 304 | 84 |
| Observed mean (SD) | 53.3 (11.3) | 50.7 (10.9) | 48.2 (10.8) |
| Month 6 |  |  |  |
| n | 574 | 271 | 67 |
| Observed mean change (SD) | -1.5 (10.4) | -1.7 (9.5) | -3.3 (8.3) |
| LS mean change (SE) | NA | NA | NA |
| Body Weight |  |  |  |
| Baseline |  |  |  |
| n | 612 | 304 | 84 |
| Observed mean (SE) | 95.5 (20.2) | 86.7 (17.5) | 90.7 (16.6) |
| Month 6 |  |  |  |
| n | 544 | 271 | 67 |
| Observed mean (SE) | 92.8 (20.1) | 83.6 (17.5) | 89.0 (16.1) |
| LS mean (SE) | 92.3 (0.2) | NA | NA |
| Change from Baseline |  |  |  |
| Observed mean change (SD) | -3.0 (3.7) | -3.3 (3.3) | -1.9 (3.4) |
| LS mean change (SE) | -3.0 (0.2) | -3.3 (0.2) | NA |
| A1c |  |  |  |
| Baseline |  |  |  |
| n | 612 | 303 | 84 |
| Observed mean (SD) | 8.6 (0.9) | 8.1 (1.1) | 7.9 (0.8) |
| Month 6 |  |  |  |
| n | 547 | 302 | 67 |
| Observed mean (SD) | 7.0 (1.1) | 6.9 (1.0) | 6.9 (0.8) |
| LS mean (SE) | 7.0 (0.04) | 6.9 (0.05) | NA |
| Change from Baseline |  |  |  |
| Observed Change | -1.6 (1.1) | -1.3 (1.0) | -1.1 (0.9) |
| LS mean change (SE) | -1.6 (0.04) | -1.2 (0.05) | NA |

DBP, diastolic blood pressure; LS, least squares; SBP, systolic blood pressure; SD, standard deviation; SE, standard error.

**Table S4. Systolic blood pressure:** **Mediation meta-analysis of placebo-controlled trials**

|  | |  |  | **Weight-dependent**  **(mmHg)** | |  | **Weight-independent**  **(mmHg)** | |  | **Total Effect**  **(mmHg)** | |  |  | **% Weight independent ^§^** |
| --- | --- | --- | --- | --- | --- | --- | --- | --- | --- | --- | --- | --- | --- | --- |
| **Study** | | **Time Point** | **N** | **Estimate**  **(95% CI)** | **p-value** |  | **Estimate**  **(95% CI)** | **p-value** |  | **Estimate**  **(95% CI)** | **p-value** |  |  |  |
| **Dula 1.5 mg vs. PBO** | |  |  |  |  |  |  |  |  |  |  |  |  |  |
| AWARD-1 | | Week 26 | 374 | -1.5 (-2.8, -0.5) | 0.005 |  | -1.6 (-4.4, 1.1) | 0.251 |  | -3.1 (-5.8, -0.6) | 0.021 |  |  | 52% |
| AWARD-5 | | Month 6 | 409 | -1.3 (-2.2, -0.7) | < 0.001 |  | -1.2 (-3.4, 1.2) | 0.307 |  | -2.5 (-4.9, -0.1) | 0.024 |  |  | 46% |
| AWARD-8 | | Week 24 | 270 | -0.3 (-1.2, 0.1) | 0.249 |  | 0.7 (-2.9, 3.7) | 0.690 |  | 0.3 (-3.2, 3.3) | 0.849 |  |  | NA |
| AWARD-10 | | Week 24 | 268 | -0.9 (-1.9, -0.2) | 0.023 |  | -2.4 (-5.2, 0.5) | 0.073 |  | -3.3 (-6.1, -0.4) | 0.015 |  |  | 72% |
| Ferdinand et al. | | Week 26 | 417 | -1.2 (-1.8, -0.5) | < 0.001 |  | -2.0 (-4.0, 0.0) | 0.049 |  | -3.2 (-5.1, -1.2) | 0.002 |  |  | 63% |
|  | |  |  |  |  |  |  |  |  |  |  |  |  |  |
| Meta-analysis [1] | |  |  | -0.94 (-1.40, -0.49) | < 0.001 |  | -1.45 (-2.60, -0.31) | 0.013 |  | -2.63 (-3.76, -1.49) | < 0.001 |  |  | 64% |
|  | |  |  |  |  |  |  |  |  |  |  |  |  |  |
| Post hoc sensitivity [2] | |  |  | -1.18 (-1.60, -0.76) | <0.001 |  | -1.78 (-3.01, -0.55) | 0.005 |  | -3.01 (-4.22, -1.80) | <0.001 |  |  | 61% |
|  | |  |  |  |  |  |  |  |  |  |  |  |  |  |
|  | **^§^**: % Weight-Independent was calculated as (1 - Weight-dependent Effect / Total Effect) * 100% and was reported only when total effect p-value < 0.05 or when the weight-dependent and weight-independent effects had the same sign.  [1]: Random-effect meta-analysis on all listed individual studies.  [2]: Random-effect meta-analysis on all listed individual studies excluding AWARD-8.  CI, confidence interval; dula, dulaglutide; NA, not applicable, PBO, placebo. | | | | | | | | | | | | | |

**Table S5. Pulse pressure: Mediation meta-analysis of placebo-controlled trials**

| **Study** |  | **N** | **Weight-dependent**  **(mmHg)** | |  | **Weight-independent**  **(mmHg)** | |  | **Total Effect**  **(mmHg)** | |  | **% Weight-independent^§^** |
| --- | --- | --- | --- | --- | --- | --- | --- | --- | --- | --- | --- | --- |
|  |  |  | **Estimate**  **(95% CI)** | **p-value** |  | **Estimate**  **(95% CI)** | **p-value** |  | **Estimate**  **(95% CI)** | **p-value** |  |  |
| **Dula 1.5 mg vs. PBO** |  |  |  |  |  |  |  |  |  |  |  |  |
| AWARD-1 | Week 26 | 374 | -0.4 (-1.3, 0.4) | 0.325 |  | -2.4 (-4.8, -0.3) | 0.026 |  | -2.8 (-5.1, -0.7) | 0.006 |  | 87% |
| AWARD-5 | Month 6 | 409 | -0.7 (-1.3, -0.2) | 0.011 |  | -0.9 (-2.8, 1.0) | 0.311 |  | -1.7 (-3.6, 0.3) | 0.063 |  | 56% |
| AWARD-8 | Week 24 | 270 | -0.1 (-0.8, 0.1) | 0.366 |  | -0.7 (-4.3, 2.5) | 0.639 |  | -0.8 (-4.3, 2.4) | 0.569 |  | 83% |
| AWARD-10 | Week 24 | 268 | -0.4 (-1.1, 0.0) | 0.092 |  | -2.6 (-4.9, -0.2) | 0.021 |  | -3.0 (-5.2, -0.6) | 0.008 |  | 87% |
| Ferdinand et al. | Week 26 | 417 | -0.5 (-1.0, 0.0) | 0.030 |  | -2.7 (-4.3, -1.2) | <0.001 |  | -3.2 (-4.8, -1.7) | <0.001 |  | 84% |
|  |  |  |  |  |  |  |  |  |  |  |  |  |
| Meta-analysis [1] | Month 6/ Week26 |  | -0.35 (-0.60, -0.11) | 0.005 |  | -2.00 (-2.96, -1.04) | < 0.001 |  | -2.5 (-3.46, -1.54) | <0.001 |  | 86% |
|  |  |  |  |  |  |  |  |  |  |  |  |  |
| Post hoc sensitivity [2] | Month 6/ Week26 |  | -0.51 (-0.82, -0.20) | 0.001 |  | -2.14 (-3.15, -1.12) | < 0.001 |  | -2.68 (-3.69, -1.66) | <0.001 |  | 81% |
|  |  |  |  |  |  |  |  |  |  |  |  |  |
| **^§^**: % Weight-Independent was calculated as (1 - Weight-dependent Effect / Total Effect) * 100% and was reported only when total effect p-value < 0.05 or when the weight-dependent and weight-independent effects had the same sign.  [1]: Random effect meta-analysis on all listed individual studies.  [2]: Random effect meta-analysis on all listed individual studies excluding AWARD-8.  CI, confidence interval; dula, dulaglutide; PBO, placebo. | | | | | | | | | | | | |

**Table S6. Diastolic blood pressure: Mediation meta-analysis of placebo-controlled trials**

| **Study** |  | **N** | **Weight-dependent**  **(mmHg)** | |  | **Weight-independent**  **(mmHg)** | |  | **Total Effect**  **(mmHg)** | |  | **% Weight-independent^§^** |
| --- | --- | --- | --- | --- | --- | --- | --- | --- | --- | --- | --- | --- |
|  |  |  | **Estimate**  **(95% CI)** | **p-value** |  | **Estimate**  **(95% CI)** | **p-value** |  | **Estimate**  **(95% CI)** | **p-value** |  |  |
| **Dula 1.5 mg vs. PBO** |  |  |  |  |  |  |  |  |  |  |  |  |
| AWARD-1 | Week 26 | 374 | -1.1 (-1.9, -0.5) | 0.003 |  | 0.7 (-1.1, 2.6) | 0.457 |  | -0.4 (-2.0, 1.4) | 0.671 |  | NA |
| AWARD-5 | Month 6 | 409 | -0.6 (-1.1, -0.3) | 0.006 |  | -0.3 (-1.5, 1.0) | 0.680 |  | -0.9 (-2.2, 0.4) | 0.186 |  | 68% |
| AWARD-8 | Week 24 | 270 | -0.2 (-0.7, 0.1) | 0.274 |  | 1.3 (-1.0, 3.4) | 0.225 |  | 1.1 (-1.1, 3.4) | 0.304 |  | NA |
| AWARD-10 | Week 24 | 268 | -0.5 (-1.2, -0.1) | 0.037 |  | 0.3 (-1.5, 2.3) | 0.758 |  | -0.2 (-2.0, 1.7) | 0.792 |  | NA |
| Ferdinand et al. | Week 26 | 417 | -0.7 (-1.1, -0.3) | 0.001 |  | 0.8 (-0.5, 2.1) | 0.212 |  | 0.1 (-1.2, 1.4) | 0.850 |  | NA |
|  |  |  |  |  |  |  |  |  |  |  |  |  |
| Meta-analysis [1] | Month 6/ Week26 |  | -0.55 (-0.80, -0.30) | <0.001 |  | 0.45 (-0.30, 1.19) | 0.239 |  | -0.22 (-0.96, 0.52) | 0.555 |  | NA |
| Post hoc sensitivity [2] | Month 6/ Week26 |  | -0.67 (-0.91, -0.43) | <0.001 |  | 0.33 (-0.46, 1.13) | 0.413 |  | -0.37 (-1.15, 0.41) | 0.355 |  | NA |
|  |  |  |  |  |  |  |  |  |  |  |  |  |
| **^§^**: % Weight-Independent was calculated as (1 - Weight-dependent Effect / Total Effect) * 100% and was reported only when total effect p-value < 0.05 or when the weight-dependent and weight-independent effects had the same sign.  [1]: Random effect meta-analysis on all listed individual studies.  [2]: Random effect meta-analysis on all listed individual studies excluding AWARD-8.  CI, confidence interval; dula, dulaglutide; PBO, placebo. | | | | | | | | | | | | |

**Table S7. Diastolic blood pressure: Indirect comparison of dulaglutide 4.5mg vs placebo**

| **Study** |  | **N** | **Weight-dependent**  **(mmHg)** | |  | **Weight-independent**  **(mmHg)** | |  | **Total Effect**  **(mmHg)** | |  | **% Weight-independent*** |
| --- | --- | --- | --- | --- | --- | --- | --- | --- | --- | --- | --- | --- |
|  |  |  | **Estimate**  **(95% CI)** | **p-value** |  | **Estimate**  **(95% CI)** | **p-value** |  | **Estimate**  **(95% CI)** | **p-value** |  |  |
| **Dula 4.5 mg vs. DULA 1.5 mg** |  |  |  |  |  |  |  |  |  |  |  |  |
| AWARD-11 | Week 26 | 1103 | -0.3 (-0.6, -0.2) | <0.001 |  | 0.6 (-0.2, 1.5) | 0.183 |  | 0.3 (-0.6, 1.2) | 0.556 |  | NA |
|  |  |  |  |  |  |  |  |  |  |  |  |  |
| **Dula 1.5 mg vs. PBO** |  |  |  |  |  |  |  |  |  |  |  |  |
| AWARD-5 | Month 6 | 409 | -0.6 (-1.1, -0.3) | 0.006 |  | -0.3 (-1.5, 1.0) | 0.680 |  | -0.9 (-2.2, 0.4) | 0.186 |  | 32% |
| Ferdinand et al. Subset | Week 26 | 128 | -0.5 (-1.2, 0.1) | 0.102 |  | 0.9 (-1.4, 3.1) | 0.442 |  | 0.2 (-1.9, 2.6) | 0.761 |  | NA |
| Meta-analysis [1] |  |  | -0.57 (-0.90, -0.24) | <0.001 |  | 0.05 (-1.2, 1.11) | 0.938 |  | -0.55 (-1.71, 0.60) | 0.348 |  | NA |
|  |  |  |  |  |  |  |  |  |  |  |  |  |
| **Dula 4.5 mg vs. PBO** |  |  |  |  |  |  |  |  |  |  |  |  |
|  |  |  |  |  |  |  |  |  |  |  |  |  |
| Indirect comparison (primary) [2] |  |  | -0.90 (-1.34, -0.46) | <0.001 |  | 0.30 (-1.28, 1.88) | 0.710 |  | -0.60 (-2.18, 0.98) | 0.457 |  | NA |
| Indirect comparison (sensitivity) [3] |  |  | -0.87 (-1.25, -0.49) | <0.001 |  | 0.65 (-0.75, 2.04) | 0.365 |  | -0.25 (-1.65, 1.14) | 0.722 |  | NA |
|  |  |  |  |  |  |  |  |  |  |  |  |  |
| *: % Weight-Independent was calculated as Weight-independent Effect / Total Effect * 100% and was reported only when total effect p-value < 0.05 or when the weight-dependent and weight-independent effects had the same sign  [1]: Calculated from random effect meta-analysis on AWARD-5 and Ferdinand et al. Subset.  [2]: Indirect comparison was performed between AWARD-11 and AWARD-5.  [3]: Indirect comparison was performed between AWARD-11 and the meta-analysis from AWARD-5 and Ferdinand et al. subset.  CI, confidence interval; Dula, dulaglutide; PBO, placebo. | | | | | | | | | | | | |

**Table S8. Systolic blood pressure: Indirect comparison of dulaglutide 4.5mg vs placebo**

| **Study** |  | **N** | **Weight-dependent**  **(mmHg)** | |  | **Weight-independent**  **(mmHg)** | |  | **Total Effect**  **(mmHg)** | |  | **% Weight-independent*** |
| --- | --- | --- | --- | --- | --- | --- | --- | --- | --- | --- | --- | --- |
|  |  |  | **Estimate**  **(95% CI)** | **p-value** |  | **Estimate**  **(95% CI)** | **p-value** |  | **Estimate**  **(95% CI)** | **p-value** |  |  |
| **Dula 4.5 mg vs. Dula 1.5 mg** |  |  |  |  |  |  |  |  |  |  |  |  |
| AWARD-11 | Week 26 | 1103 | -0.7 (-1.1, -0.4) | <0.001 |  | -0.3 (-1.6, 1.0) | 0.671 |  | -1.0 (-2.2, 0.3) | 0.147 |  | 29% |
|  |  |  |  |  |  |  |  |  |  |  |  |  |
| **Dula 1.5 mg vs. PBO** |  |  |  |  |  |  |  |  |  |  |  |  |
| AWARD-5 | Month 6 | 409 | -1.3 (-2.2, -0.7) | <0.001 |  | -1.2 (-3.4, 1.2) | 0.307 |  | -2.5 (-4.9, -0.1) | 0.024 |  | 46% |
| Ferdinand et al. Subset | Week 26 | 128 | -0.9 (-2.0, 0.1) | 0.088 |  | -2.1 (-5.7, 1.5) | 0.248 |  | -3.1 (-6.7, 0.5) | 0.096 |  | 70% |
| Meta-analysis [1] |  |  | -1.1 (-1.8, -0.5) | <0.001 |  | -1.5 (-3.4, 0.5) | 0.139 |  | -2.7 (-4.6, -0.7) | 0.007 |  | 55% |
|  |  |  |  |  |  |  |  |  |  |  |  |  |
| **Dula 4.5 mg vs. PBO** |  |  |  |  |  |  |  |  |  |  |  |  |
| Indirect comparison (primary) [2] |  |  | -2.00 (-2.88, -1.12) | <0.001 |  | -1.50 (-4.22, 1.22) | 0.280 |  | -3.50 (-6.22, -0.78) | 0.012 |  | 43% |
| Indirect comparison (sensitivity) [3] |  |  | -1.84 (-2.57, -1.12) | <0.001 |  | -1.78 (-4.17, 0.61) | 0.145 |  | -3.68 (-6.07, -1.29) | 0.003 |  | 50% |
|  |  |  |  |  |  |  |  |  |  |  |  |  |
| *: % Weight-Independent was calculated as Weight-independent Effect / Total Effect * 100% and was reported only when total effect p-value < 0.05 or when the weight-dependent and weight-independent effects had the same sign  [1]: the meta-analysis was calculated from random effect analysis on AWARD-5 and Ferdinand et al. Subset.  [2]: Indirect comparison was performed between AWARD-11 and AWARD-5.  [3]: Indirect comparison was performed between AWARD-11 and the meta-analysis from AWARD-5 and Ferdinand et al. subset.  CI, confidence interval; Dula, dulaglutide; PBO, placebo. | | | | | | | | | | | | |

**Table S9. Pulse pressure: Indirect comparison of dulaglutide 4.5mg vs placebo**

|  |  |  | **Weight-dependent**  **(mmHg)** | |  | **Weight-independent**  **(mmHg)** | |  | **Total Effect**  **(mmHg)** | |  | **% Weight-Independent^§^** |
| --- | --- | --- | --- | --- | --- | --- | --- | --- | --- | --- | --- | --- |
| **Study** | **Time Point** | **N** | **Estimate**  **(95% CI)** | **p-value** |  | **Estimate**  **(95% CI)** | **p-value** |  | **Estimate**  **(95% CI)** | **p-value** |  |  |
| **Dula 4.5 mg vs. DULA 1.5 mg** |  |  |  |  |  |  |  |  |  |  |  |  |
| AWARD-11 | Week 26 | 1103 | -0.4 (-0.6, -0.2) | 0.001 |  | -0.9 (-1.8, 0.2) | 0.112 |  | -1.2 (-2.2, -0.1) | 0.022 |  | 70% |
|  |  |  |  |  |  |  |  |  |  |  |  |  |
| **Dula 1.5 mg vs. PBO** |  |  |  |  |  |  |  |  |  |  |  |  |
| AWARD-5 | Month 6 | 409 | -0.7 (-1.3, -0.2) | 0.011 |  | -0.9 (-2.8, 1.0) | 0.311 |  | -1.7 (-3.6, 0.3) | 0.063 |  | 56% |
| Ferdinand et al. Subset | Week 26 | 128 | -0.4 (-1.1, 0.3) | 0.290 |  | -3.0 (-6.0, 0.0) | 0.053 |  | -3.4 (-6.4, -0.4) | 0.026 |  | 88% |
|  |  |  |  |  |  |  |  |  |  |  |  |  |
| Meta-analysis [1] |  |  | -0.6 (-1.1, -0.1) | 0.014 |  | -1.6 (-3.5, 0.4) | 0.108 |  | -2.2 (-3.9, -0.6) | 0.008 |  | 73% |
|  |  |  |  |  |  |  |  |  |  |  |  |  |
| **Dula 4.5 mg vs. PBO** |  |  |  |  |  |  |  |  |  |  |  |  |
| Indirect comparison (primary) [2] | Month 6/ Week26 |  | -1.10 (-1.72, -0.48) | < 0.001 |  | -1.80 (-3.99, 0.39) | 0.107 |  | -2.90 (-5.09, -0.71) | 0.009 |  | 62% |
| Indirect comparison (sensitivity) [3] | Month 6/ Week26 |  | -0.99 (-1.50, -0.48) | <0.001 |  | -2.48 (-4.64, -0.32) | 0.025 |  | -3.42 (-5.33, -1.52) | <0.001 |  | 71% |
|  |  |  |  |  |  |  |  |  |  |  |  |  |
| **^§^**: % Weight-Independent was calculated as (1 - Weight-dependent Effect / Total Effect) * 100% and was reported only when total effect p-value < 0.05 or when the weight-dependent and weight-independent effects had the same sign  [1]: the meta-analysis was calculated from random effect analysis on AWARD-5 and Ferdinand et al. Subset.  [2]: Indirect comparison was performed between AWARD-11 and AWARD-5.  [3]: Indirect comparison was performed between AWARD-11 and the meta-analysis from AWARD-5 and Ferdinand et al. subset.  CI, confidence interval; DULA, dulaglutide; PBO, placebo. | | | | | | | | | | | | |
